# Minimizing Oxidative Stress in the Lens: Alternative Measures for Elevating Glutathione in the Lens to Protect against Cataract

**DOI:** 10.3390/antiox13101193

**Published:** 2024-10-01

**Authors:** Julie C. Lim, Lanpeng Jiang, Natasha G. Lust, Paul J. Donaldson

**Affiliations:** 1 Department Physiology, University of Auckland, Auckland 1023, New Zealand; kim.jiang@auckland.ac.nz (L.J.); natasha.lust@gmail.com (N.G.L.); p.donaldson@auckland.ac.nz (P.J.D.); 2 Aotearoa New Zealand National Eye Centre, University of Auckland, Auckland 1023, New Zealand

**Keywords:** glutathione, cataracts, Nrf2, cystine/cysteine, lens microcirculation system

## Abstract

Oxidative stress plays a major role in the formation of the cataract that is the result of advancing age, diabetes or which follows vitrectomy surgery. Glutathione (GSH) is the principal antioxidant in the lens, and so supplementation with GSH would seem like an intuitive strategy to counteract oxidative stress there. However, the delivery of glutathione to the lens is fraught with difficulties, including the limited bioavailability of GSH caused by its rapid degradation, anatomical barriers of the anterior eye that result in insufficient delivery of GSH to the lens, and intracellular barriers within the lens that limit delivery of GSH to its different regions. Hence, more attention should be focused on alternative methods by which to enhance GSH levels in the lens. In this review, we focus on the following three strategies, which utilize the natural molecular machinery of the lens to enhance GSH and/or antioxidant potential in its different regions: the NRF2 pathway, which regulates the transcription of genes involved in GSH homeostasis; the use of lipid permeable cysteine-based analogues to increase the availability of cysteine for GSH synthesis; and the upregulation of the lens’s internal microcirculation system, which is a circulating current of Na^+^ ions that drives water transport in the lens and with it the potential delivery of cysteine or GSH. The first two strategies have the potential to restore GSH levels in the epithelium and cortex, while the ability to harness the lens’s internal microcirculation system offers the exciting potential to deliver and elevate antioxidant levels in its nucleus. This is an important distinction, as the damage phenotypes for age-related (nuclear) and diabetic (cortical) cataract indicate that antioxidant delivery must be targeted to different regions of the lens in order to alleviate oxidative stress. Given our increasing aging and diabetic populations it has become increasingly important to consider how the natural machinery of the lens can be utilized to restore GSH levels in its different regions and to afford protection from cataract.

## 1. Introduction

While cataract surgery is an effective treatment for restoring vision, cataract still remains a leading cause of visual impairment and remains the most significant cause of global blindness [1,2]. The burden of cataract treatment is high. The increase in the aging and diabetic populations means that cataract surgery is the most commonly performed surgical procedure in the world, inevitably placing huge strains on hospital systems and creating lengthy waiting times for cataract surgery. It has been predicted that delaying the onset of cataract by ten years will halve its incidence [1], highlighting the importance of investigating alternative ways by which to prevent or delay cataract. Oxidative stress is known to play a major role in the development of cataracts due to aging, diabetes and following vitrectomy surgery. As such, the use of antioxidant supplements has been advocated as a therapeutic approach to slow cataract progression. However, studies into their efficacy are mixed [3,4] and, in some cases and in other tissues, antioxidant supplementation has been shown to have pathological effects [5]. Accumulating evidence suggests that, rather than being harmful, physiological levels of reactive oxygen species (ROS) may in fact be beneficial, acting as signalling molecules that are important for maintaining normal cellular processes [6,7,8]. In support of this, Chen et al. showed that low levels of ROS can initiate redox signalling and cell proliferation in human lens epithelial cells [9], for the first time demonstrating a physiological role for ROS in the growth and development of the lens that is not linked to cataract formation. Unwittingly, antioxidant supplementation may suppress “good” ROS, resulting in dysregulated ROS signalling and disease progression. Hence, a better understanding is required of the delicate balance between ROS and the antioxidant defences in the body that allow ROS to exercise their physiological role without causing damage to cells.

Exogenous antioxidants that might have strong antioxidant potential have been tested for their abilty to delay or prevent cataract (reviewed by [10]). However, the results have been mixed and are most likely due, in part, to the inabilty of the lens to uptake and accumulate these “foreign” compounds. On the other hand, endogenous antioxidants would have more favourable chances of uptake as the lens would contain the natural molecular machinery to uptake these compounds. However, the AREDS2 clinical trial showed that supplementation of endogenous antioxidants, such as lutein/zeaxanthin, ascorbic acid (vitamin C), and vitamin E, had no overall effect on the need for cataract surgery [11]. Little is known about how lutein/zeaxanthin and vitamin E is transported into the lens and, while ascorbic acid transporters have been identified in the lens [12], human lenses lack the ability to synthesise ascorbic acid due to the lack of the enzyme catalysing the final step of the biosynthetic pathway [13]. On the other hand, our group and others have shown that the lens utilises a number of different ways to uptake, synthesise and regenerate glutathione (GSH) (reviewed in [14]).

Glutathione is the principal antioxidant in the lens, existing in millimolar concentrations and has been shown to be a vital antioxidant critical for maintaining transparency in young lenses [15,16,17,18]. With diabetes and advancing age, GSH levels are known to be differentially depleted in different lens regions. In the lens cortex, oxidative modifications to transporters/channels impair the ability of the fibre cells to maintain their cell volume, resulting in the morphological damage seen in diabetic cortical cataract. Furthermore, oxidative modifications in the lens nucleus or core result in the improper crosslinking of proteins in central mature fibre cells that manifest as age-related nuclear cataract (reviewed in [19]). Hence, by tapping into the natural machiney of the lens, we have hypothesized that it may be possible to harness its natural machinery to restore GSH levels either through enhanced uptake, synthesis or regeneration when it becomes depleted. To implement this strategy it has become apparent that a better understanding of the different mechanisms utilised to regulate GSH metabolism in the different regions of the lens is required to restore GSH levels and prevent the specific damage phenotypes associated with either diabetic cortical cataract or age-related nuclear cataract.

In this review, we will briefly discuss the structural adaptations of the lens that result in differences in the ways in which the lens accumulates GSH in its different regions. We will then detail what happens to GSH levels as a consequence of aging, diabetes and following vitrectomy surgery, and then highlight strategies that could be utilized to enhance GSH levels in the different regions of the lens. These include the Nrf2 pathway which regulates the transcription of genes involved in GSH synthesis and regeneration in the lens cortex, the use of lipid-permeable, cysteine-based analogues to increase cysteine levels and GSH synthesis in the lens cortex and finally the upregulation of the lens microcirculation system as a means of specifically enhancing the delivery of GSH or potentially cysteine-based analogues to increase antioxidant potential in the lens nucleus (core). While much more research is required to address the issues around drug delivery to the lens, it is hoped that a better understanding of ROS, antioxidants and lens physiology will help to shape effective strategies by which to enhance GSH levels and reduce the cataract burden caused by the increasingly aged and diabetic population.

## 2. Structural and Metabolic Adaptations within the Lens to Preserve Transparency

**Figure 1:**
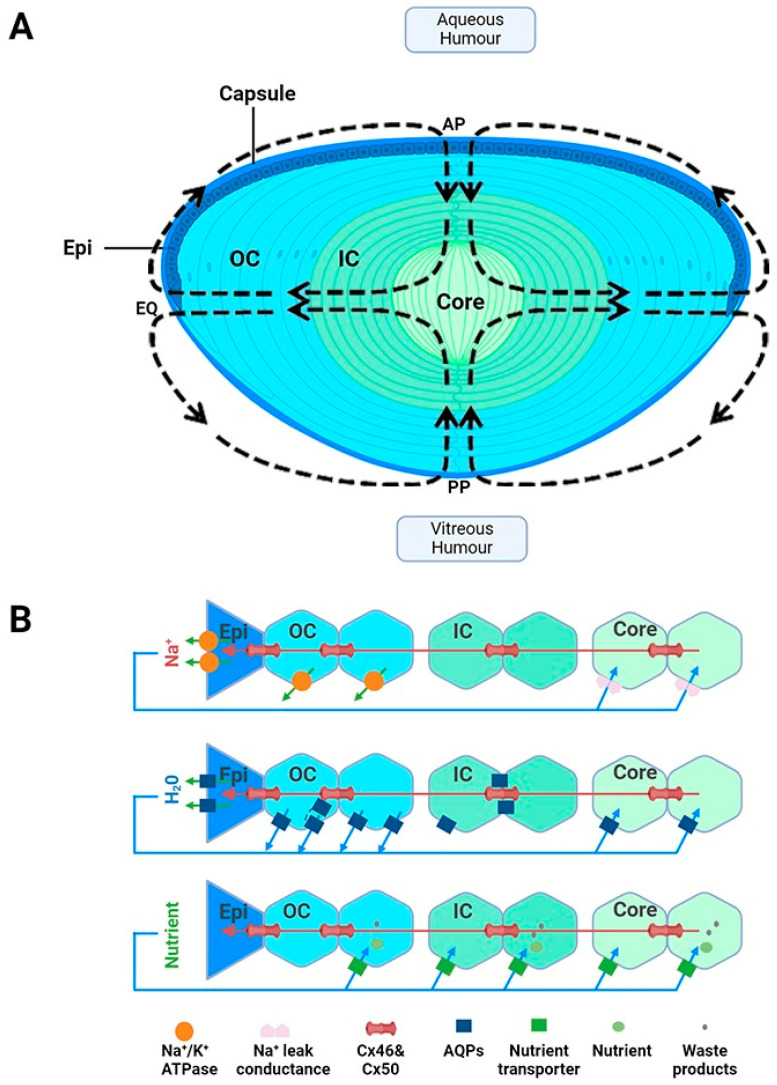
Lens structure. (**A**): Architecture of the lens showing the capsule, anterior epithelial monolayer (Epi), elongating nucleated outer cortical fibre cells (OC), anucleated inner cortical fibre cells (IC) and mature fibre cells in the lens core (C). Fibre cells from adjacent hemispheres meet at the anterior (AP) and posterior (PP) poles to form the sutures. Arrows in the diagram represent the direction of ion and water fluxes that underpin the lens microcirculation system. (**B**): Top panel—an equatorial cross section of the lens showing a cellular view of ion movement in the lens. Current and solutes are proposed to flow into the lens via the extracellular space, to cross fibre cell membranes, and to flow outward via a gap-junction-mediated pathway (Cx 56 and Cx 50) at the lens equator (EQ) where the Na^+^/K ATPase pumps are concentrated. Middle panel—solute movement results in water crossing the membranes via aquaporin (AQP) water channels, which possess different water permeabilities and exhibit regional differences in their expression, subcellular localization, and regulation, thereby differentially contributing to water influx, outflow, and efflux, respectively. Bottom panel—isotonic fluid flux delivers nutrients to the lens nucleus, where they are accumulated by nutrient uptake transporters. Unwanted metabolites or waste products can then be removed from the lens. Adapted from [37] with permission from Elsevier.

Suspended within the anterior chamber of the eye, the lens serves to precisely and dynamically focus light onto the retina. In addition to this optical role, the lens also acts as a site of synthesis, storage and delivery of antioxidants. In this role, the lens is used to protect not only itself from oxidative stress, but also surrounding tissues through its ability to export antioxidants into the ocular humours. Structurally, the lens is enveloped by a capsule, which is a thickened basement membrane composed of collagen type IV fibrils and is intertwined within a matrix composed of glycosaminoglycans and glycoproteins (Figure 1A) [20]. A monolayer of epithelial cells is found beneath the capsule and covers the surface of the anterior lens (Figure 1A). Differentiation, proliferation and elongation of epithelial cells take place in the germinative zone, situated just above the lens equator (EQ) eventually leading to the formation of fibre cells, which constitutes the majority of the lens [21]. These cells withdraw from the cell cycle and elongate anteriorly and posteriorly, migrating towards the central core and forming layers of concentric shells (Figure 1A). Due to the continuous mitotic division of fibre cells, an age gradient is established whereby there is displacement of older fibre cells located in the lens core by newly formed, differentiating fibre cells found in the lens cortex [22]. The cells are well coupled by an extensive network of gap junctions containing Cx46 and Cx50 connexins [23], which provides a low resistance pathway for diffusion between cells so as to form a metabolic syncytium [24,25].

To ensure the effective transmission of light from the lens to the retina, the lens contains several structural adaptations. The lens is avascular as the presence of haem pigments in blood would result in the absorption of light rather than its transmission to the retina [21]. The fibre cells are arranged in a highly organised manner, with fibre cells in the outer cortex exhibiting a flattened hexagonal cross-sectional morphology [26]. This facilitates tight packing that minimises extracellular space and creates a diffraction lattice that minimises light scattering [27]. As these cortical fibre cells differentiate and become internalised, they degrade their light-scattering organelles, such as mitochondria, nuclei and endoplasmic reticulum. While the absence of these cellular structures minimises the dispersion of light, it also means that mature fibre cells lack the ability to synthesise new protein and rely on anaerobic metabolism for their energy requirements. The inability to make new proteins in these deeper lying mature fibre cells means that this region of the lens is particularly susceptible to oxidative damage, as there is no mechanism to replace damaged proteins. Additionally, there are changes in the expression of cytoplasmic and membrane proteins, particularly the soluble cytoplasmic crystallins. These proteins are mainly expressed in the nucleus of the lens and are densely packed to ensure a smooth radial gradient of refractive index from the cortex to the core (reviewed in [19]).

While these structural adaptions establish lens transparency, the absence of a blood supply means that the lens requires a specialised system to ensure that the cellular structure and solubility of the crystallin proteins critical for lens transparency are maintained. To achieve this the lens generates a unique microcirculation system that utilises circulating fluxes of ions and water to maintain fibre cell volume and to deliver essential nutrients and antioxidants specifically to the lens’s centre, which, due to the size of the lens, is not something that can be achieved by passive diffusion alone [28,29,30]. This microcirculation system is driven by an internally circulating ionic current that enters at both anterior and posterior surfaces and exits at the equator of the lens (Figure 1A). At the poles, the inward flow of Na^+^ through the extracellular spaces causes water to flow, and with it essential nutrients such as antioxidants, glucose and amino acids, deep into the lens centre [30]. A cross-sectional view of the lens demonstrates how Na^+^ diffuses through the extracellular spaces, crosses fibre cell membranes and then then flows toward the surface using an intercellular pathway mediated by gap junction channels (Figure 1B, top panel). These gap junctions direct Na^+^ fluxes toward the lens periphery, where Na^+^ is actively removed by Na^+^-K^+^-ATPase concentrated at the lens equator (Figure 1B panel). The inward flow of Na^+^ through the extracellular spaces causes water to flow (Figure 1B, middle panel), and with it essential nutrients such as antioxidants, glucose and amino acids (Figure 1B, bottom panel), deep into the lens’s centre [30]. Membrane transporters specific for the uptake of glucose, cysteine and glycine [31,32,33,34,35,36] have been shown to be expressed in this region, thus facilitating the uptake of these nutrients, which are delivered to this region by the microcirculation system. In summary, in the absence of a blood supply, this microcirculation system is essential for driving the transport of ions and water and the delivery of nutrients required to ensure that the optical and transparent properties of the lens are maintained (reviewed in [19]). With all of these structural and metabolic adaptations in mind, it is important to consider how this may affect GSH delivery, synthesis and regeneration in the different regions of the lens.

## 3. Regional Differences in GSH Uptake, Synthesis, Export and Regeneration

Glutathione is the principal antioxidant in the lens, existing in high concentrations [15,16,17,18]. This is necessary given the essential functions of GSH in the lens, including the elimination of reactive oxygen species, preserving protein thiols in a reduced form, regenerating the antioxidant ascorbic acid, serving as a co-factor for repair and antioxidant enzyme systems, and participating in the elimination of reactive xenobiotic compounds and/or endogenous metabolites via GSH conjugation [15,16,17,18,38,39,40,41]. GSH levels in the lens exist at ~4–6 mM, which is higher than any other ocular tissue [41], being around nine times higher than that in the cornea [42]. The levels of GSH in the lens are also higher than the kidney (2–3 mM) and as high as those found in the liver (5–7 mM), which are traditionally thought to play an active role in regulating GSH levels in the plasma [43]. The high levels of GSH in the lens is predicted to have a function in not only protecting the lens from oxidative damage but also through its ability to export GSH and/or supply GSH precursor amino acids to neighbouring avascular tissues like the cornea and trabecular meshwork and thereby minimise oxidative stress to these tissues. This has been supported by the studies undertaken by Kinoshita and Masurat, which have shown that bovine lenses cultivated in an isotonic phosphate buffer demonstrate the release of GSH over a four-hour period [44] and that rat lenses, and more recently human donor lenses, cultured under iso-osmotic conditions were also able to release GSH [45,46]. Moreover, studies of the rat lens have shown that, in the presence of acivcin, a substance that suppresses the activity of the enzyme responsible for breaking down GSH, gamma-glutamyl transpeptidase (GGT), resulted in an increase in the release of GSH [45]. This suggests that the lens might serve as a source of not only GSH but also the amino acids that serve as precursors for GSH to other tissues of the eye, thereby protecting neighbouring tissues from oxidative stress.

**Figure 2:**
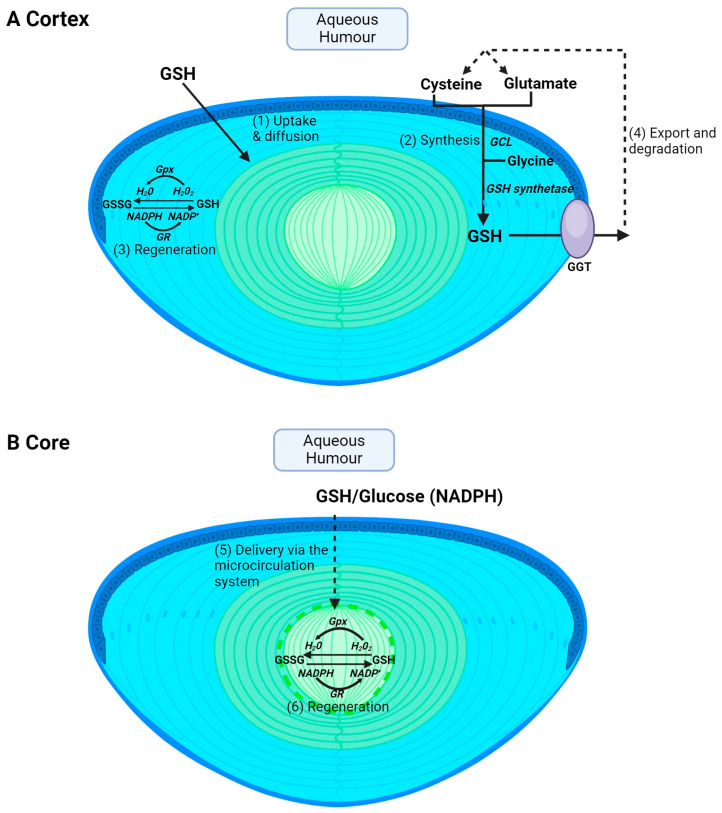
Regional differences in GSH accumulation in the lens. The different structural adaptations of the lens mean that the cortex and core accumulate GSH via different pathways. (**A**) In the cortex, GSH can be taken up directly from the aqueous humour via GSH transporters and then GSH can be supplied to cortical fibres via diffusion through gap junctions (1). Within cortical fibre cells, GSH can also be synthesised intracellularly from precursor amino acids, cysteine, glutamate, and glycine, by the sequential actions of the enzymes glutamate cysteine ligase (GCL) and glutathione synthetase (2). GSH can also be regenerated from GSSG. GSH is used as a co-factor for glutathione peroxidase (Gpx) in the process of detoxifying H_2_O_2_ into H_2_O and is in turn oxidised to GSSG. Glutathione reductase (GR) is used in combination with NADPH to help recycle GSSG back to GSH (3). Finally, GSH can be exported from the lens followed by degradation into its precursor amino acid by GGT and subsequent reuptake of amino acids for GSH synthesis (4). (**B**) In the core, passive diffusion is insufficient to deliver GSH to the lens’s centre. Instead, the microcirculation delivers GSH to the core where it can then be taken up into cells via GSH transporters (5). GSH levels can also be maintained by GSSG regeneration (6) but cannot be synthesised in the core.

Glutathione is obtained from the diet through the consumption of sulphur-rich proteins found in cruciferous vegetables and meats such as beef, fish and poultry [47]. However, extracellular GSH is rapidly degraded into its constituent amino acids, and GSH levels in the aqueous and vitreous humours are ~2 μM [48] and ~100 μM [49], respectively, in humans. The lens utilises a number of pathways to accumulate GSH. However, the pathways used by the cortex (Figure 2A) versus the core (Figure 2B) will differ due to regional differences in GSH delivery, metabolism and synthesis in the different regions of the lens. In the lens cortex, GSH can be directly taken up from the ocular humours [50,51,52] (Figure 2A—pathway 1), but the identity of transporters that mediate this uptake remain unknown. The lens can also accumulate GSH via uptake of its precursor amino acids, cysteine, glutamate and glycine, followed by intracellular synthesis via the sequential actions of GCL and GSH synthetase (Figure 2A—pathway 2). Transporters that uptake cysteine, glutamate and glycine have been identified in rat and human lenses [32,33], along with a transporter for the uptake of cystine in rat, mouse and human lenses [34,35,36]. Cysteine can also be synthesized intracellularly via the trans-sulphuration pathway, in which homocysteine is converted to cystathionine and subsequently to cysteine [53]. Fan et al. showed that, in an LEGSKO mouse in which de novo GSH synthesis was completely abolished, lens GSH levels were reduced by up to 60% [54], highlighting the important role of GSH synthesis in maintaining GSH levels in the mouse lens. The lens cortex is also able to regenerate GSH from oxidised GSH (GSSG) by GSH reductase (GR) and the co-factor NADPH generated through the metabolism of glucose via the pentose phosphate pathway (Figure 2A—pathway 3). Finally, GSH can also be exported from the lens and then broken down into its constituent amino acids via GGT (Figure 2A—pathway 4), thereby supplying the aqueous humour with an additional pool of amino acids that can be taken up and recycled to increase intracellular GSH levels. However, in the core, not all of these pathways can be used to accumulate GSH (Figure 2B). GSH synthesis is restricted to the metabolically active epithelium and cortex. This generates a gradient of GSH, with the highest levels occurring in the epithelium and cortical fibre cells and the lowest in the lens nucleus [41,55,56]. This suggests that a GSH concentration gradient exists in the lens that drives the delivery of GSH to the nucleus via passive diffusion through gap junction channels. However, the calculated rates of delivery of GSH from the cortex to the nucleus by passive diffusion alone are too slow and it has thus been proposed that the lens microcirculation system (Figure 2B—pathway 5) mediates the delivery of GSH to these deeper lying fibre cells. Moreover, there are differences in GR activity, with higher levels in the cortex relative to the nucleus [57]. However, the level of GR activity in the nucleus is sufficient to drive GSH regeneration if NADPH is provided (Figure 2B—pathway 6). These differences between the lens cortex and core in the accumulation of GSH are an important consideration towards developing strategies in ensuring that GSH levels are restored in the appropriate region of the lens. This in turn will afford protection against the oxidative stress that induces the distinctly different cataract phenotypes observed in the cortex and core of the lens.

## 4. Oxidative Stress and Cataract Risk

The damage phenotypes of age-related nuclear cataract and diabetic cortical cataract are distinctly different. Age-related cataract typically results in opacification of the lens centre or nucleus of the lens with no obvious signs of damage to fibre cell structure, while, in contrast, diabetic cataracts appear as opacifications in the lens cortex caused by disruption to lens fibre cell volume, which distorts the morphology of the lens cortex. Increased oxidative stress is well recognized as a major contributor to the pathogenesis of age-related nuclear cataract [18], while increasing evidence shows that not only osmotic stress, but also oxidative stress, contributes to diabetic cataract formation [19,58]. In addition, cataracts induced following vitrectomy have also been shown to be linked to increased oxygen levels and oxidative stress [59,60]. Because oxidative stress is a common thread in the initiation of cataracts, despite their different damage types, we have summarized the literature below to determine what is known about GSH and its contribution to oxidative stress and cataracts due to age, diabetes and following vitrectomy surgery.

### 4.1. Age-Related Nuclear (ARN) Cataract

ARN cataracts are characterized by the gradual clouding of the central part of the lens, and severity is based on the degree of brunesence and opalescence in the central region [61]. In patients with ARN cataract, the occurrence of nuclear cataract is linked to the reduction in sulfhydryl groups in lens proteins, where more than 90% of cysteine residues and approximately 50% of methionine residues are oxidized [62,63,64]. Consequently, there is an increase in the formation of mixed disulfides between proteins and an elevation in the proportion of water-insoluble proteins [65]. This results in the enhanced formation of disulfide bonds between proteins and other cross-linkages, leading to the aggregation of proteins and the scattering of light. Numerous studies have extensively examined these biochemical changes [16,18] and there is a consensus that the decrease in GSH levels and excessive oxidative stress are the primary factors contributing to the development of ARN cataracts.

Multiple explanations for why GSH levels deplete with age in the lens nucleus have been advanced [66], and it is likely that a combination of events contribute to cataract formation. Slavi has demonstrated that GSH is able to be delivered to the core through diffusion from the cortex via gap junctions [67], albeit at low levels. GSH levels were found to be signficantly reduced in the nucleus of Cx46 KO mice, but not Cx50 KO mice, suggesting that GSH is able to diffuse to the nucleus via Cx46 gap junction channels [67]. In the young lens, low GSH permeability via Cx46 channels is proposed to be offset by high levels of coupling conductance. However, with aging, coupling conductance decreases by ~70% in the deeper fibre cells [68]. The authors propose that this reduction in coupling, in combination with decreased GSH synthesis in the outer cortex, would limit GSH diffusion from the outer cortex to the core leading to an observed depletion in GSH levels in the core with age [68]. Truscott has also proposed that an intracellular barrier forms around middle age, which would also explain the limited diffusion and depletion of GSH into the lens core [69]. An alternative view is that GSH is delivered to the core through an internal microcirculation system that generates a flux of ions and water that circulates through the lens (Figure 1) [19,28,37]. With age, it has been proposed that the efficiency of this circulation system decreases, potentially reducing GSH delivery and uptake into the lens core [19,37]. While both views can specifically explain the depletion of GSH in the core, recent work has strengthened the idea of the involvement of the lens microcirculation system.

### 4.2. Diabetic Cortical Cataract

Diabetes is characterized by long term hyperglycaemia due to abnormalities in either insulin secretion, insulin function, or both [70]. There are many types of diabetes, wherein the prevalent varieties are type 1 and type 2 diabetes. Type 1 diabetes, commonly identified in children, may also present in adults as a more gradual variant. This condition is distinguished by the autoimmune destruction of pancreatic β cells responsible for insulin production, leading to an insufficient insulin supply. In contrast to type 2 diabetes, individuals with type 1 diabetes are usually not overweight, and there exists a hereditary inclination for autoimmune destruction in pancreatic β cells [71]. Type 2 diabetes, often identified in middle-aged individuals, exhibits a hereditary element, although the likelihood escalates with elevated body mass index. This condition is distinguished by the presence of insulin resistance and inadequate response from the pancreatic β cells, leading to a deficiency of insulin.

Cataract is a common complication observed in people diagnosed with type 1 or type 2 diabetes. Patients with diabetes are more susceptible to developing cataracts, with a 2–5 times higher risk, and are prone to experiencing cataracts at a younger age [72]. It has been reported that there is a five-fold increase in the risk of diabetic cortical cataract compared with nondiabetics [73]. Moreover, the formation of cataract occurs at a younger age and progresses more rapidly in individuals with diabetes in comparison with those without diabetes [74,75]. The incidence of cortical and/or posterior subcapsular cataracts (PSC) is higher in diabetic patients compared with non-diabetics. Although other forms of cataract can and do present in diabetic patients, cortical cataracts tend to be the more prevalent form [75].

While a depletion of GSH in the lens nucleus is associated with age-related nuclear cataract formation, increased oxidative stress is also associated with diabetic cortical cataract [76,77,78]. Diabetic cataract is associated with the accumulation of sorbitol, an impermeable osmolyte, produced because of excess glucose. This results in osmotic stress and cell swelling in the lens cortex. As diabetic cataract can take years to manifest, it has been proposed that, over time, accumulated oxidative stress results in modifications of the cell volume machinery of the lens. In support of this, oxidative stress manifests early on as a depletion of glutathione in the human diabetic lens [79]. In this case, depletion of GSH is most likely due to a decrease in the availability of NADPH, due to its consumption when glucose is converted into sorbitol [73]. This depletion of NADPH means that GSH cannot be regenerated from GSSG by glutathione reductase. Other signs of increased oxidative stress in the diabetic lens includes the accumulation of lipid peroxidation products, such as malondialdehyde [77], an increase in GSSG, depletion of other antioxidants such as ascorbate and taurine [80], and a decrease in the activity of antioxidant enzymes such as superoxide dismutase, catalase and glutathione peroxidase [76,78]. It is now recognised that osmotic and oxidative stress both contribute to diabetic cataract formation [58,81], and that, in particular, chronic oxidative stress impairs the osmoregulatory mechanism of the lens. While our laboratory has identified volume regulatory transporters and their associated regulatory kinases and phosphatases in the rat, bovine and human lenses [82,83,84], further work is required to show how oxidative stress modifies the function of these transporters and regulatory proteins.

### 4.3. Cataracts Post Vitrectomy

Cataract development may also arise following ocular surgery. Vitrectomy is a commonly performed surgery in which the gel-like vitreous is removed from the back of the eye and replaced with saline. It is typically performed in order to access and repair the diseased retina. However, while vitrectomy is essential for the treatment of multiple retinal disorders, a consequence of vitrectomy is that it accelerates the progression of cataracts, resulting in the need for cataract surgery within two years [85,86,87]. The demand for vitrectomy has also significantly increased in the past years, especially with the increase of the aging and diabetic populations. A recent New Zealand study has revealed that the total number of vitrectomies increased by 50% between the years 2009–2018, with the number of patients aged 65 years or older undergoing vitrectomy surgery increasing by 91% [88].

The molecular mechanism is most likely attributed to loss of the gel-like barrier which limits the diffusion of oxygen and exposure of the lens to oxygen and/or the loss of the antioxidant ascorbic acid which is normally present at high concentrations in the vitreous humour, where it acts to consume oxygen [59,60]. In support of this, a gradient of oxygen exists in the vitreous chamber, where there is a gradual decline in oxygen levels from the retina towards the lens [89]. Oxygen levels were around ~22 mmHg close to the retina, ~7 mmHg in the central vitreous and ~9 mmHg close to the posterior side of the lens. However, following vitrectomy, this oxygen gradient is lost, and oxygen levels increase to ~13 mmHg in the central vitreous and ~14 mmHg close to the lens. It has also been shown that patients post-vitrectomy contained ~40% lower ascorbic levels compared with patients with no previous vitrectomy [60]. This suggests that, following vitrectomy, ascorbic levels are never fully restored and are insufficient to keep oxygen levels low in the vitreous. Over time, it is predicted that elevated oxygen levels in the vitreous will eventually manifest as cataracts requiring surgery. This might suggest that replenishment of the vitreous chamber with ascorbic acid may be a strategy by which to help reduce oxygen levels and protect the lens from cataract. Interestingly, young patients that develop cataracts post vitrectomy tend to present with posterior subcapsular cataract, while patients over 50 are more likely to develop nuclear cataracts [90]. GSH levels have never been measured in these lenses, which might suggest that the mechanism of cataract formation in older patients may be an accelerated form of ARN cataract. If this is the case, GSH supplementation may also be beneficial in protecting the lens from cataract formation following vitrectomy. In vitro studies using human lens epithelial cells have demonstrated that the addition of GSH significantly improves the stability of ascorbic acid [91], suggesting that a combination of the two antioxidants may perhaps play an important role in delaying cataracts post vitrectomy.

## 5. Challenges of GSH Supplementation to the Lens

Because oxidative stress and loss of GSH homeostasis are characteristic inducers of cataract formation, a relatively simple strategy would be to supplement the lens with GSH. High-dose oral antioxidant supplements are used by many with the intent to prevent or slow the progression of ARN cataract. However, the human gastrointestinal tract contains significant amounts of the enzyme GGT which may prevent significant intact absorption of GSH from oral supplementation. Because of this, only a few human clinical trials have evaluated the effects of oral GSH supplementation. Witschi et al. administered a single oral dose of up to 3 g of GSH to seven healthy subjects and did not observe an increase in blood GSH levels after 4.5 h [92]. Allen et al., evaluated the impact of longer term, oral GSH supplementation as part of a randomized, double-blinded, placebo-controlled trial in which healthy, adult humans were given 500 mg GSH twice daily for 4 weeks. However, no significant changes were observed in either biomarkers of oxidative stress or in glutathione status [93]. This would suggest that oral glutathione supplementation would be unlikely to enhance glutathione levels in the aqueous humour or to be of any therapeutic benefit to the lens.

Another strategy would be to apply glutathione directly as an eye drop. However, a prospective study, in which topical glutathione (2% reduced GSH) was applied to the eye four times a day for one month, and, in some patients with age-related cataracts, for three months, revealed no beneficial effects, with no significant change in cataract parameters [94]. In retrospect this is not surprising, as physiological barriers in the eye, including the tears, the cornea and the blood–aqueous barrier as well as the intracellular and extracellular barriers of the lens, all make topical drug delivery challenging, with these issues not just associated with GSH but any drug administered by topical delivery to the eye. Other approaches aim to increase GSH levels by application of drugs that are believed to boost GSH synthesis or focus on the delivery of cysteine and its derivatives as this is the limiting factor of the glutathione synthesis [95,96]. All of these strategies, however, also have to deal with the general problem of inefficient topical ocular drug delivery.

While intra-ocular injections are invasive in some situations, this might be acceptable for some patients. For example, injection of a dose of GSH/ascorbic acid during vitrectomy to prevent cataract formation might be a strategy for boosting antioxidant protection post-surgery. However, while this might be beneficial initially, over time, frequent administration of injectable antioxidants may be required to protect the lens. In regard to this, the field of drug delivery systems for the eye has advanced, with a focus on sustained-release drug delivery systems to avoid repeatable injections for diseases such as age-related macular degeneration, diabetic retinopathy and retinal vein occlusion. However, there are challenges with developing sustained release drugs and mitigating adverse effects such as inflammation, intraocular pressure increases, and corneal issues due to implant migration. These topics are outside the scope of this review but reviews in this area have been widely published [97,98]. Advancements in the field will most likely ensure that, in due course, these types of systems will be widely adopted when efficacy and safety has been established through large-scale trials.

## 6. Alternative Strategies to Enhance Endogenous GSH Levels in the Lens

In addition to improving the stability, bioavailability and delivery of compounds to the lens, it is equally important to consider how these compounds can be used to influence GSH levels in the different regions of the lens. Any therapies aimed at restoring GSH levels to the lens cortex or nucleus need to consider where GSH is taken up in the lens and how it is subsequently delivered to and utilized by the lens centre. In this section, we will explore strategies that utilise the endogenous machinery of the lens to synthesise and deliver GSH and/or its precursor amino acid cysteine or derivatives of cysteine to the different regions of the lens.

### 6.1. The Nrf2 Pathway

Nuclear factor erythroid 2-related factor 2 (Nrf2) is a transcription factor involved in buffering oxidation-reduction status in cells [99,100]. Ubiquitously expressed, Nrf2 has been linked to many processes, including regulation of cellular stress responses, drug metabolism and defence against carcinogenesis and inflammatory insults [101,102,103,104]. Importantly, Nrf2 functions as a major regulator of cellular resistance to oxidants, controlling 20 antioxidant genes [105], some of which involve the regulation of GSH homeostasis through synthesis and regeneration of GSH [106,107].

**Figure 3:**
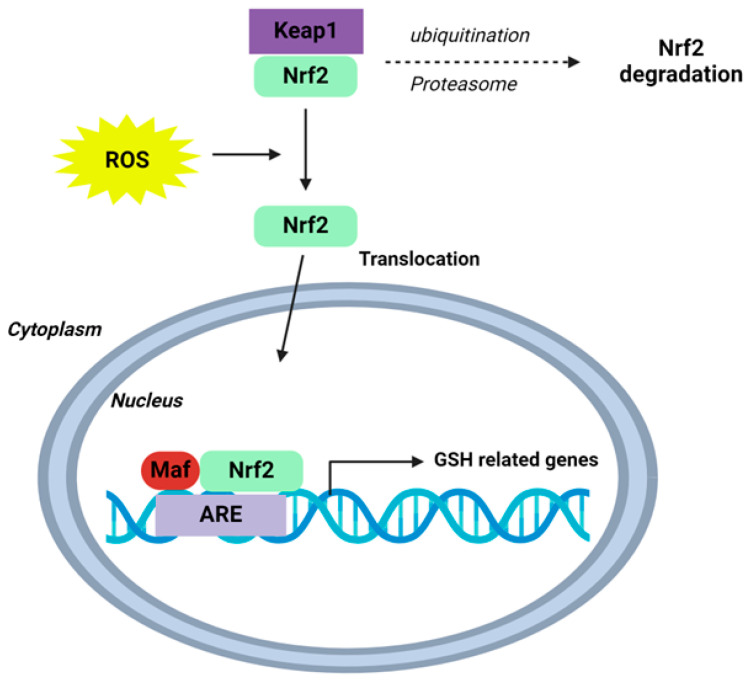
Schematic of the Nrf2-Keap1-ARE signalling pathway. Under normal conditions, nuclear erythroid-2 like factor-2 (Nrf2) is ubiquitinated through Kelch-like ECH-associated protein1 (Keap1) and degraded in the proteasome. After ROS exposure, Keap1 is inactivated and Nrf2 translocates to the nucleus and binds to antioxidant response element (ARE) sites, subsequently activating many genes, including those involved in GSH homeostasis. Schematic adapted from [118]. Creative Commons Licence CCBY 4.0.

Under physiological conditions, cytosolic Nrf2 is bound to Kelch-like ECH-associated protein1 (Keap1), targeting Nrf2 for ubiquitination and proteasome-dependent degradation, therefore supressing Nrf2 activity. Following oxidative stress, modification of Keap1 cysteine residues allows for Nrf2 to escape ubiquitination and translocate to the nucleus [108,109,110,111,112,113]. Here, Nrf2 binds antioxidant response elements (AREs) on DNA, activating the transcription of enzymes which promote GSH synthesis—glutamate–cysteine ligase catalytic (GCLC) and modulator (GCLM) subunits—as well as regenerating glutathione synthetase (GS) and GSH (Figure 3) [114,115]. In turn, de novo GSH is produced in the cytosol, replenishing stores and cellular antioxidant capacity [116].

Given the link between Nrf2 and GSH, it is unsurprising that researchers have investigated the involvement of Nrf2 in preventing cataract formation in animal and human lenses. Rowan et al. examined the development of cataracts in Nrf2 knockdown mice and revealed that male and female KO mice developed cataracts at 11–15 months, which were not apparent in age-matched wild type C57BL/6J mice [117]. KO mice were observed to develop different types of cataracts, including cortical cataracts, posterior subcapsular cataract and nuclear cataract, with cortical and posterior subcapsular cataracts the more common types [117]. Histological analysis of 18-month WT and KO mice revealed that, in the KO lenses, there were disruptions in cell architecture in the cortex, with the abnormal presence of nuclei in deeper regions of the lens and ectopic nuclei in the posterior region [117]. Taken together, these findings highlight the importance of the Nrf2 pathway in lens development and cataract formation.

A number of studies have investigated the ability of Nrf2 to delay or prevent cataracts using animal-based cataract models. Administration of sodium selenite via intraperitoneal injection in pups is a popular cataract model as it rapidly induces calcium-dependent oxidative stress, reduces GSH levels and protein insolubility, and results in nuclear opacification of the lens within 4–6 days [119,120,121,122,123,124]. Palsamy et al. have shown that selenite suppresses Nrf2/Keap-dependent antioxidant protection through DNA demethylation of the Keap1 promoter, resulting in the overexpression of Keap1 and suppression of Nrf2 [125]. Fang et al. have reported that selenite-induced opacification was delayed when rats were treated with the antioxidant trimetazidine (TMZ). TMZ was shown to inhibit demethylation of the Keap1 promoter, resulting in enhanced Nrf2 expression levels [126]. Ferulic acid (FA) is a phenolic compound found in several Chinese medicines and edible plants and has been found to have antioxidative and radioprotective effects by activating Nrf2 [127]. Rats exposed to ionizing radiation displayed histological alterations to lens epithelial cells, along with increased markers of oxidative stress and reduced GSH levels and GR activity in whole lenses [127]. However, administration of FA to rats for four consecutive days prior to ionising radiation and then three consecutive days after, showed that histological alterations were reduced by FA [127]. Moreover, increased nuclear translocation of Nrf2 was increased, along with increased expressions of GCLC and GR, and an increase in GSH levels [127]. H_2_O_2_-induced opacification in cultured porcine lenses was reported to be reduced by pre-treatment with sulforaphane (SFN), a dietary isothiocyanate and an activator of Nrf2 [128]. Similarly, H_2_O_2_-induced opacification in mice lenses was shown to be reduced by hydralazine, an FDA-approved vasodilator drug, that has been shown to enhance the Nrf2/ARE-mediated antioxidant gene and protein expression [129]. Overall, these studies provide a range of evidence supporting the idea of a role for Nrf2 in protection against cataract. However, many of these animal models do not necessarily replicate the aetiology and the slow, time-dependent failure of antioxidant mechanisms seen in age-related cataract and diabetic cataract.

Human studies offer insight into the translational potential of these models. Human lens epithelial cells (HLECs), collected from subjects of varying ages, have been shown to demonstrate a marked increase in Keap1 gene expression, with increasing age associated with the demethylation of its promoter region and reduced Nrf2 expression [130]. A similar finding has also been reported from the cataractous lenses of diabetic patients, with significant levels of demethylated DNA in the Keap1 promoter compared with clear lenses [131]. Various suppressors of Nrf2 can mimic features of cataracts in HLECs, such as apoptosis, endoplasmic reticulum stress and oxidative stress, while activators of Nrf2 have been shown to protect HLECs from features of cataract that are induced under treatment with homocysteine or hydrogen peroxide [125,132,133,134]. Yang et al. have reported that the culturing of HLECs in homocysteine induces the demethylation of Keap promoter DNA, activating the expression of Keap1 and increasing the targeting of Nrf2 for proteosomal degradation. This results in the reduced expression of catalase, superoxide dismutase, gluathione peroxidase and GSH [135]. However, in the presence of acetyl-l-cartinine, significant protection from these effects has been reported [135]. In cultured HLECs, sestrin 2 afforded protection from H_2_O_2_-induced oxidative stress and apoptosis by activating the Nrf2 pathway. Liu et al. showed that, when HLECs are exposed to H_2_O_2_-induced oxidative stress, cells pre-treated with SFN are found to be more resistant to cytotoxicity, apoptosis and DNA strand breaks [128]. This is a result of increased Nrf2 signalling through its translocation and accumulation in the nucleus and the upregulation of antioxidant genes, including GCLM. SFN has also been used to activate Nrf2 and modulate the expression of the antioxidant gene, peroxiredoxin 6 (Prdx6) in HLECs [136,137]. However, it has also been found that higher doses of SFN (>6 μM) activate death signalling by the repression of *Prdx6* expression, which increases reactive oxygen species (ROS) load and cell death and thus indicates that SFN has a narrow window where it is beneficial.

While there is evidence of the utility of Nrf2 in increasing antioxidant pathways in the lens and in minimising oxidative stress, Nrf2 activation and increased GSH synthesis/antioxidant protection might be limited to the lens epithelial and cortical fibre cells and so alternative strategies are required to alleviate oxidative stress and replenish GSH levels in the lens centre.

### 6.2. The Use of Cysteine Analogues

Cysteine is the rate-limiting amino acid required for GSH synthesis. In addition, the sulfhydryl group of cysteine acts as a potent reducing moiety, giving cysteine antioxidant properties. Therefore, it is important to recognise the different pathways used by the lens to accumulate cysteine, not only for its ability to synthesise GSH but also to potentially act as an antioxidant itself. Cysteine can be synthesised de novo via the transulfuration pathway, in which methionine is metabolised to homocysteine and then the conversion of cysteine through cystathionine. In human and porcine lenses, Persa et al., identified the rate-limiting enzyme that converts homocysteine to cystathionine, cystathionine-beta-synthase (CBS), and found that the greatest intensity was found in the epithelial layer, with lower but equal quantities of CBS detected in the cortical and nuclear regions [53]. In human epithelial cells and porcine lenses, H_2_O_2_ was able to upregulate the gene expression of CBS, indicating that, under oxidative stress, the transulfuration pathway can be upregulated to drive the conversion of homocysteine to cysteine for GSH production and provide additional antioxidant potential for the cells [53]. Our laboratory has also identified transporters involved in the direct uptake of cysteine in the lens and showed that alanine serine cysteine transporter 2 (ASCT2) is expressed in the rat [33] and human lens [35], with expression of ASCT2 evident in both the cortex and nucleus. However, under low pH conditions, ASCT2 preferentially uptakes glutamate and, since the lens core has a lower pH compared with the lens cortex [138], it is unclear whether ASCT2 mediates cysteine uptake in this region of the lens.

Cystine, the oxidised form of cysteine, is more stable than the free amino acid cysteine, and, in the aqueous humour, the more oxidising conditions favour a predominance of cystine over cysteine [139] and over GSH or GSSG [48]. The uptake of cystine may therefore be a more relevant mechanism for the physiological accumulation of cysteine into the lens. In other tissues, cystine uptake is mediated by the cystine/glutamate exchanger (referred to as System xc^−^) which imports cystine into the cell in exchange for glutamate. Upon intracellular accumulation, cystine is rapidly reduced to cysteine, where it can subsequently be used for GSH synthesis [140]. We have shown that the rat and human lens express xCT, the light chain subunit of System xc^−^ (in the lens cortex and nucleus in young rat and human donor lenses [34,35]). The expression of xCT in these two regions of the lens, the outer cortex, which is capable of GSH synthesis, and the nucleus, which lacks the ability to synthesise GSH, suggests that the accumulation of cysteine is used for different purposes. In the outer cortex, cysteine would be used for GSH synthesis, while, in the nucleus, cysteine itself could act as an antioxidant. However, in older human donor lenses, while xCT was found to expressed in the outer cortex, it was absent in the nucleus, suggesting that, with age, xCT may be modified, potentially reducing its ability to uptake cystine and thus accumulate cysteine for antioxidant protection within this region [35]. Hence, bypassing this transport system to enhance cysteine accumulation in the lens may represent a strategy for enhancing antioxidant levels in the lens in order to afford protection from cataract formation.

Several studies have investigated the use of lipid-permeable analogues of cysteine, such as N-acetylcysteine (NAC) and N-acetylcysteine amide (NACA). NAC is approved as a drug around the world and is a commonly used therapeutic because of its mucolytic properties [141]. NAC is an acetylated variant of L-cysteine, which increases its bioavailability, as this modification increases the chemical stability by reducing oxidation. NAC is membrane permeable and able to bypass the ASCT system and, once inside the cell, releases cysteine to eventually form glutathione [142]. In the lens, NAC has been studied for its role in the treatment of cataracts using different animal models. Zhang et al. examined the in vivo effect of topical NAC (0.01% and 0.05%) on streptozotocin-induced diabetic rats [143]. The progression of lens opacity in the treated groups was slower than that of the untreated group in the early period but differences became insignificant after the sixth week. Aydin et al. evaluated the effects of NAC administered intraperitoneally (150 μg/g body weight) on selenite-induced nuclear cataract formation in rat pups [144], which is an acute model of cataract formation. Dense nuclear opacities were significantly reduced in the treatment group relative to the non-treatment group and correlated with increased GSH levels and decreased malondialdehyde and protein carbonyl levels, suggesting that NAC inhibits selenite-induced cataracts in the rat model by reducing oxidative stress. In another in vivo study, Fan et al. examined the effect of topical NAC (0.1%) administered twice daily and then 5 days per week starting at 2 months for 7 months of human vitamin C transporter 2 (hSVCT2) transgenic mice [145]. It was found that NAC significantly decreased protein-bound fluorescence at 335/385 nm, suggesting that NAC can block the protein-bound formation of AGE fluorescence and the potential modifications associated with age-related cataracts. Using cultured rabbit lenses, Wang et al. examined the effect of NAC (5 mM–40 mM) in preventing hyperoxia-induced lens opacification and changes to Na^+^/K^+^-ATPase activity; CAT, GR, and GSH levels; and water soluble protein content [146]. Cortical opacifications were reduced in lenses treated with 20 mM or 40 mM NAC, which was accompanied by increased Na^+^/K^+^-ATPase activity, catalase activity and water soluble protein compared with untreated lenses. GR activity remained unchanged and GSH levels were only significantly increased with 20 mM NAC. The results from these selected studies show some changes in delaying opacifications. These changes correlate with those found in some biochemical parameters; however, this is very difficult to assess because of the different animal models used, the different mode of administration of NAC and the different biochemical parameters measured. It has also been suggested that NAC’s low bioavailability (6–10%) and hydrophilicity [142] may contribute to the mixed results around the efficacy of NAC in cataract prevention.

N-acetylcysteine-amide (NACA), a derivative of N-acetylcysteine, has higher lipophilicity and membrane permeability, and can therefore be used at lower concentrations than NAC, which will reduce the adverse effects associated with the administration of higher doses of NAC [147]. Tobwala et al. have demonstrated NACA’s improved antioxidant ability compared with that of NAC in human lung and liver cells [148,149]. Several studies have investigated NACA for its antioxidant properties in the lens. Maddirala et al. examined the ability of NACA to prevent selenite-induced cataract formation in Wistar rats [95]. NACA was injected intraperitoneally on postpartum days 9, 11, and 13 and then topical NACA was administered from postpartum day 15 to 30. NACA was shown to reduce the severity of the cataract by enhancing GSH levels, inhibiting malondialdehyde, and lowering calcium levels, preventing activation of m-calpain and proteolysis of crystallin proteins. NACA has also been shown to inhibit cataracts in Wistar pups injected with L-buthionine-(S,R) sulfoximine (BSO), an inhibitor of GSH synthesis [150]. Rat pups were pretreated with NACA before the injection of BSO and then given injections of NACA (250 mg/kg body weight) every day until day 15. The effects of preventing cataract formation were marked between treatment and non-treatment groups, with only 20% of lenses in the treatment group developing a cataract, compared with 100% in the non-treatment group. In addition, GSH levels were replenished, along with reduced malondialdehyde and protein carbonyl formation and increased GR, catalase and GPx activity in the treatment group compared with the non-treatment group. These studies are promising but are limited by the small sample size, the use of very young rats, the type of administration (topical vs. IP) and the frequency of administration of NACA, and so further studies are needed to evaluate the role of NACA in the management of human cataract prevention.

More recently, attention has been drawn to ((2R, 2R′)-3,3′-disulfanediyl bis(2-acetamidopropanamide)), or diNACA, which is the immediate precursor in the synthesis of NACA [151]. Hector et al. compared the potential of NACA and diNACA in human cystinosis fibroblast cell cultures as potential treatments for cystinosis [152]. NACA displayed more rapidity and greater potency in cystine-depleting activity than diNACA. Although the exact mechanism of the anticystinotic activity of NACA and diNACA requires investigation, it has been suggested that NACA or diNACA may react with cystine to form a mixed disulfide as a means of reducing cystine accumulation. In the lens, the use of diNACA with increased lipophilicity would allow the lens to take up diNACA without the need to be taken up by a cystine transporter such as System xc-. Martis et al. examined the potential of diNACA or NACA to inhibit H_2_O_2_-induced cataract formation in cultured porcine and rat lenses and showed that pretreatment with either NACA or diNACA is effective in reducing H_2_O_2_-induced opacities [96]. Lenses pretreated with NACA followed by H_2_O_2_ exposure resulted in increased cysteine levels in the lens cortex and increased GSH levels in the epithelium and cortex, showing that NACA can increase the bioavailability for cysteine, in turn resulting in increased GSH synthesis and protection of the lens from H_2_O_2_-induced cataract [96]. On the other hand, pretreatment with diNACA did not increase the bioavailability of cysteine or GSH levels in any of the regions of the rat lens when exposed to H_2_O_2_ [96]. This suggests that the ability of diNACA to reduce lens opacites was not via increased GSH synthesis, but by some other mechanism which might involve mixed disulfide formation. Mixed disulfides in the lens are thought to help buffer lens proteins from further modifications [153], such as protein disulphide formation and protein aggregation, and may explain why lenses treated with diNACA were still able to reduce opacities without increasing GSH levels.

A limiting factor of many of the above studies is whether NAC, NACA or diNACA can enhance CSH or GSH levels specifically in the lens nucleus. Presumably the effects of these cysteine-based analogues would be limited to the outer cortex. This would minimise oxidative damage to the cortex, but is unlikely to have any effects on the lens centre, which is the region of the lens affected by nuclear cataracts. This raises the question as to how we can deliver these analogues to the lens centre.

### 6.3. Upregulation of the Lens Microcirculation System

The realisation that, in the absence of a blood supply, the lens generates its own unique internal microcirculation system opens the prospect that this microcirculation system can be utilised to increase the delivery of nutrients and antioxidants specifically to the lens nucleus, where they can be taken up by mature fibre cells that are known to express an array of nutrient and antioxidant transporters [31,32,33,34,35]. In this regard, we have used MRI imaging of the bovine lens organ, cultured in MRI contrast agents, as tracers of extracellular delivery, to confirm that the microcirculation system can indeed deliver solutes to the lens nucleus faster than is predicted to occur via passive diffusion alone [154]. More recently, our laboratory has used imaging mass spectrometry to visualise the time course of movement of isotopically labelled glucose ([U–^13^C], a more physiologically relevant molecule, into different regions of organ-cultured bovine lenses [155]. As expected, the initial site of glucose uptake occurred primarily in the equatorial region of the lens. However, a secondary, slower delivery of the labelled isotope to the deeper layers of the lens was also observed. This delivery of glucose to the lens nucleus occurred faster than what would be predicted via passive diffusion alone [155]. Furthermore, in addition to glucose, the imaging mass spectrometry approach was able to detect isotopically labelled glucose metabolites in the lens nucleus, suggesting that, not only was glucose delivered to the nucleus, but it was also taken up and metabolised by mature fibres in the lens nucleus. These findings are the first indication that lens microcirculation facilitates the delivery, uptake and metabolism of nutrients in the lens nucleus and supports the suggestion that upregulation of the microcirculation system could be exploited as a strategy to enhance antioxidant delivery to the lens nucleus to delay the onset and progression of age-related nuclear cataract.

**Figure 4:**
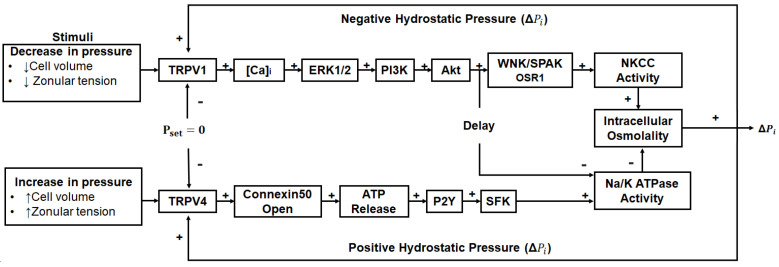
Regulation of the microcirculation system. Lens water transport (pressure) is regulated by a dual-feedback pathway. Lens surface pressure (*P_set_*) is maintained by two arms of a dual-feedback system that regulates the ion transporters that control the intracellular osmolarity of cells at the lens surface. Increases in pressure (Δ*P_i_*) due to hypoosmotic stress or increased zonular tension work via TRPV4 to activate a signalling pathway that involves the release of ATP via hemichannels, the subsequent activation of purinergic P2Y receptors, and prompts the Src family of protein tyrosine kinases (SFK) to increase the activity of the Na^+^/K^+^-ATPase and decrease lens pressure. Decreases in pressure (Δ*P_i_*), due to hyperosmotic stress or decreased zonular tension, work via TRPV1 to activate the extracellular signal-regulated kinase 1/2 (ERK1/2), phosphatidylinositol 3-kinase (PI3K)/Akt, kinase with no lysine (WNK), and Ste20-related proline–alanine-rich kinase (SPAK)/oxidative stress-responsive kinase-1 (OSR1) signalling pathways to directly activate the sodium potassium dichloride cotransporter (NKCC) and reduce the activity of the Na^+^/K^+^-ATPase to increase lens pressure. Adapted from [37] with permission from Elsevier.

This notion of upregulating the microcirculation system as a potential anti-cataract strategy has been further strengthened in recent years by the discovery that the outflow of water through lens-gap junctions (Figure 1B, middle panel) generates a substantial hydrostatic pressure gradient [156] and that this pressure gradient is highly regulated by a dual feedback system [157]. The two arms of this dual feedback pathway are modulated by the activation of the mechanosensitive ion channels TRPV1 and TRPV4 that respond to decreases and increases, respectively, in lens pressure (Figure 4). It has subsequently been shown that these changes are also reciprocally activated by changes to extracellular osmolarity [158,159,160] and by changes in the tension applied to the lens by the zonules which attach the lens to the ciliary muscle [161,162]. Hence, we envisage that this dual feedback system can be pharmacologically manipulated to upregulate water transport through the lens and therefore enhance the delivery of endogenous antioxidants and exogenous molecules designed to supplement lens antioxidant capacity in the core region of the aging lens. For example, the upregulation of the microcirculation system could be coupled with the delivery of cysteine permeable analogues described above. This would ensure that these analogues are delivered to the lens nucleus, where they could cross the lipid bilayer and so bypass the need for a membrane transporter and alleviate oxidative stress in the region of the lens most impacted by age-related nuclear cataract.

## 7. Conclusions

Given the challenges with GSH delivery to the lens and the distinct pathways of GSH accumulation in the cortex versus nucleus, alternative strategies that harness the endogenous machinery of the lens to enhance GSH levels are required. Membrane-permeable cysteine derivatives and the activation of Nrf2 show promise in their ability to increase GSH levels in the lens cortex and may be beneficial in delaying the cortical cataract typical of that seen in diabetes. However, the existence of the lens’s internal microcirculation system, which drives water transport and GSH accumulation in its nucleus, offers an exciting approach to potentially direct cysteine derivatives to the lens nucleus, which is the area most affected by aging. Further understanding of the lens’s internal microcirculation system and how it can be upregulated to enhance targeted delivery of antioxidants to the lens centre will be an important focus of future work. Overall, these approaches may open avenues for more considered strategies for developing and testing anti-cataract strategies, based on our knowledge of lens physiology, that can restore GSH levels in the lens cortex or core to maintain the optical properties and transparency of the lens and prevent the onset of cataract.

## Data Availability

No new data were created in this review, however, further inquiries can be directed to the corresponding author.
